# BO-1055, a novel DNA cross-linking agent with remarkable low myelotoxicity shows potent activity in sarcoma models

**DOI:** 10.18632/oncotarget.9657

**Published:** 2016-05-29

**Authors:** Srikanth R. Ambati, Jae-Hung Shieh, Benet Pera, Eloisi Caldas Lopes, Anisha Chaudhry, Elissa W.P. Wong, Ashish Saxena, Tsann-Long Su, Malcolm A.S. Moore

**Affiliations:** ^1^ Department of Cell Biology, Memorial Sloan Kettering Cancer Center, New York, NY, United States; ^2^ Department of Pediatrics, Memorial Sloan Kettering Cancer Center, New York, NY, United States; ^3^ Institute of Biomedical Sciences, Academia Sinica, Taipei, Taiwan

**Keywords:** Ewing sarcoma, soft-tissue sarcomas, DNA damage, DNA repair, PDX models

## Abstract

DNA damaging agents cause rapid shrinkage of tumors and form the basis of chemotherapy for sarcomas despite significant toxicities. Drugs having superior efficacy and wider therapeutic windows are needed to improve patient outcomes. We used cell proliferation and apoptosis assays in sarcoma cell lines and benign cells; γ-H2AX expression, comet assay, immunoblot analyses and drug combination studies in vitro and in patient derived xenograft (PDX) models. BO-1055 caused apoptosis and cell death in a concentration and time dependent manner in sarcoma cell lines. BO-1055 had potent activity (submicromolar IC50) against Ewing sarcoma and rhabdomyosarcoma, intermediate activity in DSRCT (IC50 = 2-3μM) and very weak activity in osteosarcoma (IC50 >10μM) cell lines. BO-1055 exhibited a wide therapeutic window compared to other DNA damaging drugs. BO-1055 induced more DNA double strand breaks and γH2AX expression in cancer cells compared to benign cells. BO-1055 showed inhibition of tumor growth in A673 xenografts and caused tumor regression in cyclophosphamide resistant patient-derived Ewing sarcoma xenografts and A204 xenografts. Combination of BO-1055 and irinotecan demonstrated synergism in Ewing sarcoma PDX models. Potent activity on sarcoma cells and its relative lack of toxicity presents a strong rationale for further development of BO-1055 as a therapeutic agent.

## INTRODUCTION

Alkylating agents are a part of the standard of care regimens for a number of pediatric and adult malignancies. Currently used alkylating drugs play a significant role in the management of soft tissue sarcomas and Ewing sarcoma, both as first-line and second-line treatments [1, 2] albeit with substantial toxicities. Therefore, there is a need for safer and less toxic alternatives that do not exhibit multi-drug cross-resistance. We have previously designed and synthesized a series of potent alkylating agents, in which the phenyl N-mustard pharmacophore is linked to the DNA-affinic molecule *via* a urea, carbamate or hydrazinecarboxamide linker to reduce the chemical reactivity of N-mustard [3–5]. To improve the water-solubility, we linked a benzene moiety with various hydrophilic side chains to the N-mustard moiety and evaluated the cytotoxicity in various cancer cell lines *in vitro* and in human xenograft models [6]. Of these agents, BO-1055 (water-soluble Ureidomustine) was found to have a broad spectrum of antitumor activity with a favorable safety profile and pharmacokinetics in pre-clinical studies [7, 8]. In this study, we evaluated its efficacy in sarcomas and performed a comprehensive toxicity screening in a range of benign cells.

**Figure 1:**
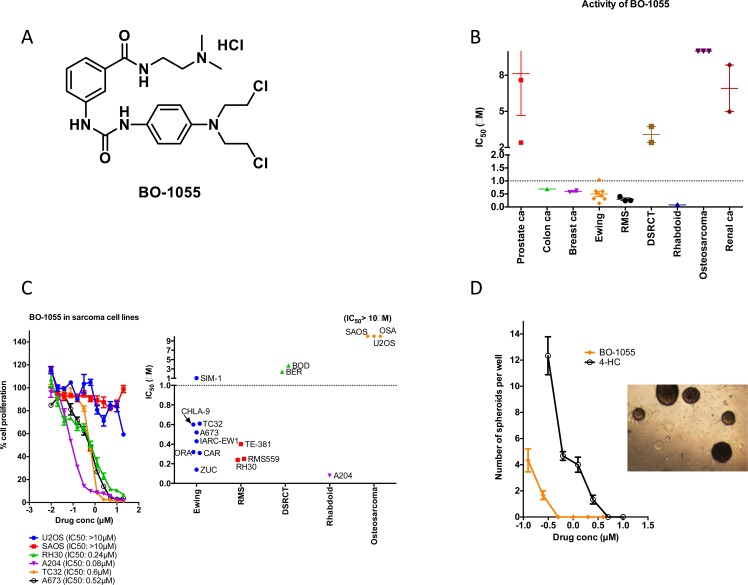
BO-1055 has potent activity in most sarcomas **A.** Structure of BO −1055. **B.** IC50 (shown on y-axis) of BO-1055 in different solid tumor cell lines. **C.** Activity of BO-1055 in sarcoma cell lines based on Alamar Blue cell proliferation assay. **D.** Spheroid assay using A673 cells in methylcellulose at various concentrations of BO-1055 and 4-HC. A representative picture of A673 spheroids in controls is shown.

BO-1055 (Figure 1A) is a bifunctional alkylating agent that is able to induce interstrand cross-links (ICLs) [4]. The potency of this class of drugs correlates with the extent of ICL formation. ICLs cause replication arrest, induction of DNA double-strand breaks and can ultimately trigger cell death [9]. Repair of ICLs was noted to be one of the prominent mechanisms of resistance to N-mustard derivatives, e. g, resistance to melphalan in multiple myeloma and chronic lymphocytic leukemia [10, 11]. There are different mechanisms involved in the repair of DNA lesions induced by specific alkylating agents and different tumors vary widely in their ability to repair such lesions [9]. DNA damage induced by BO-1055 is repaired by a number of mechanisms including nucleotide excision repair (NER), homologous recombination (HR) and O^6^-methylguanine-DNA methyltransferase (MGMT) [12]. Like melphalan, BO-1055 induces N-alkyl adducts that are repairable by NER and HR pathways. In addition, BO-1055 produces O-alkyl adducts (like BCNU/carmustine), which are repairable by MGMT [12]. Because of tumor heterogeneity, cells that evade the cytotoxic stress undergo selective expansion of resistant clones leading to treatment failure [13]. For successful elimination of all cancer cells, one has to employ multi-drug combinations that will produce diverse genomic lesions to overcome the ability of cells to escape the effects of single drug. Therefore, in this study, we evaluated the single agent activity of BO-1055 and its combination with topoisomerase I and II inhibitors, heat shock protein 90 inhibitor (PU-H71) and anthracycline (doxorubicin), based on their potential for synergism with alkylating agents. We validated our results in patient derived tumor xenograft (PDX) models that have been shown to correlate better with the antitumor activity noted in patients [14].

## RESULTS

### BO-1055 inhibits proliferation and induces cell death in different sarcoma cell lines and cultures derived from patient samples with minimal toxicity to benign cells

BO-1055 had submicromolar IC_50_ values for Ewing sarcoma, rhabdomyosarcoma cell lines and Ewing sarcoma patient samples. It had intermediate activity on DSRCT cell lines (IC_50_ = 2-3μM) and very weak activity on osteosarcoma cell lines (IC_50_ > 10μM). The activity of BO-1055 in sarcomas was evaluated and compared to that in various other cancer cell lines including lymphomas, prostate, colon, renal, breast, small cell lung cancer, myeloid and lymphoid leukemias (Figure 1B). It revealed that this agent has superior activity in Ewing sarcoma and rhabdomyosarcoma and poor activity in osteosarcoma (Figure 1B, 1C). A representative sample of growth inhibition curves for sarcoma cell lines are shown in Figure 1C with mean IC_50_ for BO-1055. We compared the anti-proliferative effect of BO-1055 and 4-HC *in vitro* (Figure 1D) at various concentrations by spheroid formation assay. As shown in Figure 1D, BO-1055 was able to inhibit A673 spheroids at a 10 fold lower concentration when compared to 4-HC. Complete inhibition of spheroids was noted at 0.5μM of BO-1055 and 5μM of 4-HC.

**Figure 2:**
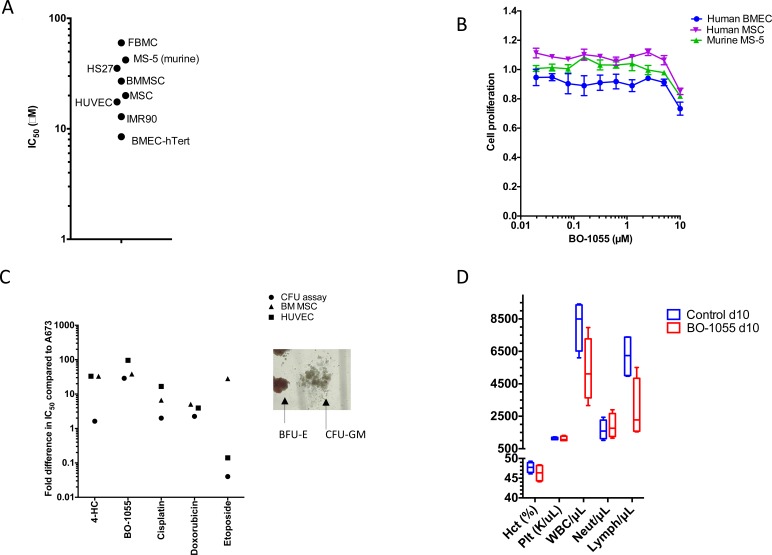
A. Toxicity of BO-1055 tested in benign primary cells or immortalized cells derived from primary cells **B.** Alamar blue assay showing cytotoxicity of BO-1055 in human and mouse mesenchymal and human endothelial cells. **C.** Comparison of BO-1055 with other DNA damaging agents including 4-Hydroxycyclophophamide (active compound of Cyclophosphamide), Doxorubicin, Cisplatin and Etoposide in benign cells. Values are represented as fold difference compared to their respective IC50 in A673. A representative picture of CFU colonies- burst-forming unit-erythroid (BFU-E) and colony-forming unit-granulocyte, macrophage (CFU-GM) is shown **D.** Hematologic parameters including hematocrit (Hct), platelets (Plt), white blood cell count (WBC), neutrophils (Neut) and lymphocytes (Lymph) in C57 healthy mice: control and B0-1055 treatment groups at d10. IMR-90- fetal lung myofibroblasts; FBMC- fetal bone marrow mesenchymal cells; HUVEC-human umbilical vein endothelial cells; HS27 and BMMSC - Adult human BM derived mesenchymal cells; BMEC-hTert- immortalized bone marrow- derived microvascular endothelial cells; MSC- human mesenchymal stromal cells, MS-5- murine mesenchymal stromal cells.

We also examined the effect of BO-1055 in inhibiting the cell growth of different types of benign cells including human mesenchymal stromal cells, fetal lung fibroblasts and myofibroblasts, hematopoietic progenitor cells, bone marrow derived endothelial cells and human umbilical vein endothelial cells and murine mesenchymal stromal cells, MS-5 (murine). Remarkably, it was revealed that BO-1055 had minimal to no cytotoxicity up to 10 μM (Figure 2A, 2B) against most of the tested cells.

### BO-1055 has a wider therapeutic window in comparison to other DNA damaging agents and chemotherapeutic agents

We tested the activity of DNA damaging agents including 4-HC, cisplatin, doxorubicin, etoposide and BO-1055 in hematopoietic progenitor cells (by colony forming unit (CFU) assay), HUVEC, human MSC and compared it to their activity in A673 cells to determine their therapeutic window. The ratio of IC50 in benign cells to that of Ewing sarcoma (A673) cells is shown for each drug. BO-1055 has one of the more favorable profiles (wider therapeutic window) in different benign cell models (Figure 2C).

### Effect of BO-1055 on hematopoietic progenitor cells

We performed the colony forming unit (CFU) assays using human CD34+ hematopoietic stem cells isolated from cord blood to elucidate hematologic toxicity. We noted no effect on BFU-E, GM and mixed colonies at a concentration < 5μM of BO-1055 on CFU assay (Figure 2C). *In vivo* toxicity testing of BO-1055 in C57 healthy mice did not reveal a significant change in hematocrit, platelet count or neutrophil count at day 10 (Figure 2D).

### BO-1055 induces cell cycle arrest in G2 phase

**Figure 3:**
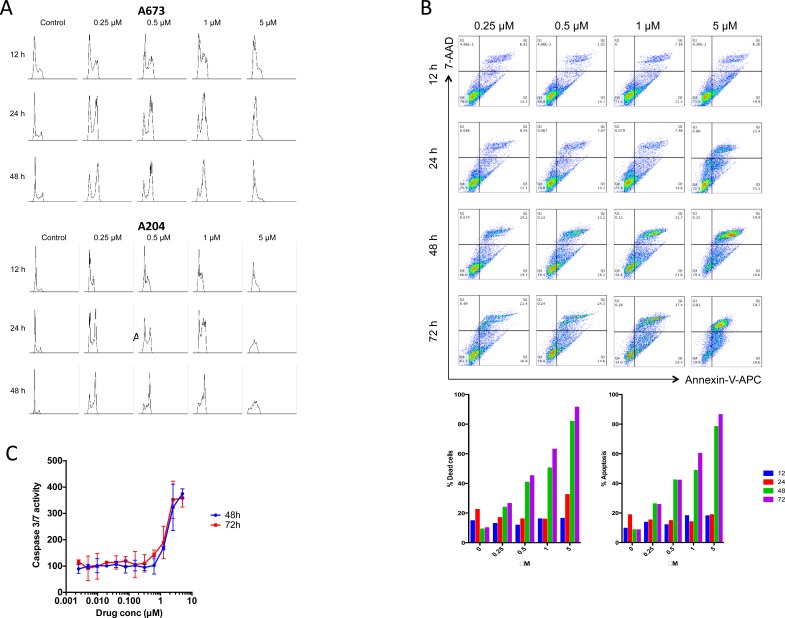
A.BO-1055 induces cell cycle arrest in G2/M phase in A673 cells and A204 cells **B.** Flow cytometric analysis of A673 cell line using 7-AAD (for viability testing) and Annexin V-APC (for apoptosis) staining at 12, 24, 48 and 72 hours at indicated concentrations of BO-1055. Also shown are the percentages of apoptotic and dead cells at different time points. **C.** Caspase 3/7 activity in A673 cells at 48h and 72h after treatment with BO-1055.

We studied the effects of BO-1055 on the cell cycle of A673 and A204 cells and found a concentration-dependent accumulation of cells in the G2/M phase (Figure 3A) that was prominent at 12-24h of exposure.

### BO-1055 induces caspase mediated cell death in sarcoma cell lines

In order to quantify the apoptosis ratio in A673 cells treated with BO-1055 we performed Annexin V-APC/7-AAD staining assays. Early apoptosis was defined as the presence of Annexin V-positive and 7-AAD-negative cells. Late apoptosis was defined as Annexin V-positive and 7-AAD-positive cells. Total percent apoptosis was defined as the sum of early and late apoptotic cells as a percentage of the total cell number. BO-1055 induces apoptosis in the A673 cell line in a time-concentration dependent manner (Figure 3B). Around 50 % of treated cells entered apoptosis after 48 h at concentrations of 1μM. Activation of caspase 3/7 in A673 cells was noted in a concentration dependent manner using ApoTox -Glo Triplex Assay (Figure 3C).

### BO-1055 induced more DNA double strand breaks and γH2AX in A673 cells compared to hMSC and cord blood CD34+ cells

**Figure 4:**
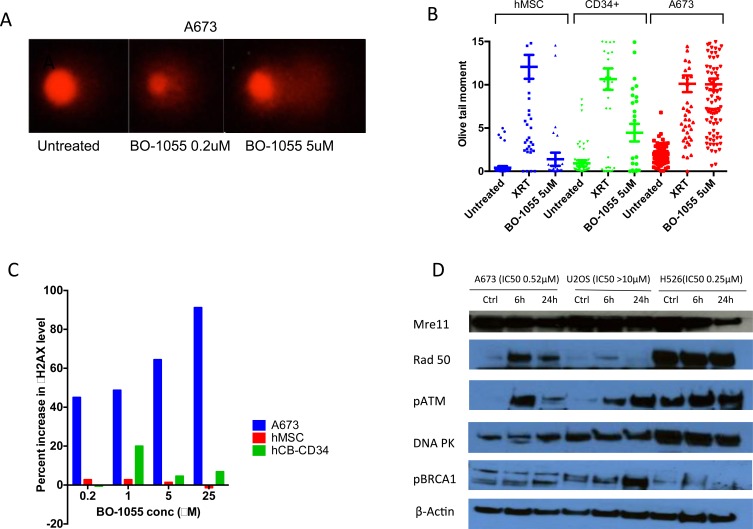
A. Comet assay showing tail formation in A673 cells up on treatment with BO-1055 for 3h **B.** Olive tail moment (micrometers) in hMSC, cord blood CD34+ hematopoietic progenitor cells and A673 cells in 3 conditions-untreated, exposed to 8Gy radiation, treatment with BO-1055 at 5μM conc. **C.** Induction of γH2AX in different cells treated with increasing concentration of BO-1055. **D.** Western blot showing induction of proteins involved in DNA damage repair in two sensitive cell lines- A673 (Ewing sarcoma) and H526 (small cell lung cancer) and a resistant cell line- U20S (osteosarcoma) treated with 2μM concentration of B0-1055 for 6h and 24h.

We used comet assay [15], which includes single cell gel electrophoresis to assess DNA breaks when exposed to radiation or DNA damaging agents. Comet assay showed increased tail size suggesting accumulation of DNA breaks in A673 cells, as the concentration of BO-1055 was increased. Olive moment (micrometers), a product of tail DNA% and the distance between the centroids of head and tail, is an accurate parameter of DNA damage. In A673 cells treated with BO-1055 and radiation (XRT) olive moment is comparable. Human MSC and cord blood CD34+ cells treated with BO-1055 have relatively less olive moment compared to XRT (Figure 4B) suggesting less toxicity of BO-1055 to the benign cells. Gamma-H2AX (γH2AX) phosphorylation is the initial step in DNA damage response following double stranded breaks and is responsible for recruiting DNA repair proteins. Assessment of γH2AX foci by flow cytometry allows assessment of DNA damage and repair [16]. γH2AX level was significantly elevated in A673 cells compared to hMSC and cord blood CD34+ cells (Figure 4C) suggesting greater DNA damage to the A673 cells when compared to benign cells.

### Induction of DNA repair proteins in cells treated with BO-1055 suggests role of multiple DNA damage response pathways

At 6h and 24h after treatment with BO-1055, we noted activation of pATM in both sensitive cell lines: A673 (Ewing sarcoma), H526 (small cell lung cancer) and resistant cell line: U20S. Upregulation of Rad50 was noted in the sensitive lines whereas upregulation of pBRCA1 was noted in U20S (Figure 4D).

### BO-1055 caused tumor regression in cyclophosphamide resistant patient-derived Ewing sarcoma xenografts and A204 (rhabdoid cell line) xenografts, and showed inhibition of tumor growth in A673 (Ewing sarcoma cell line) xenografts

**Figure 5:**
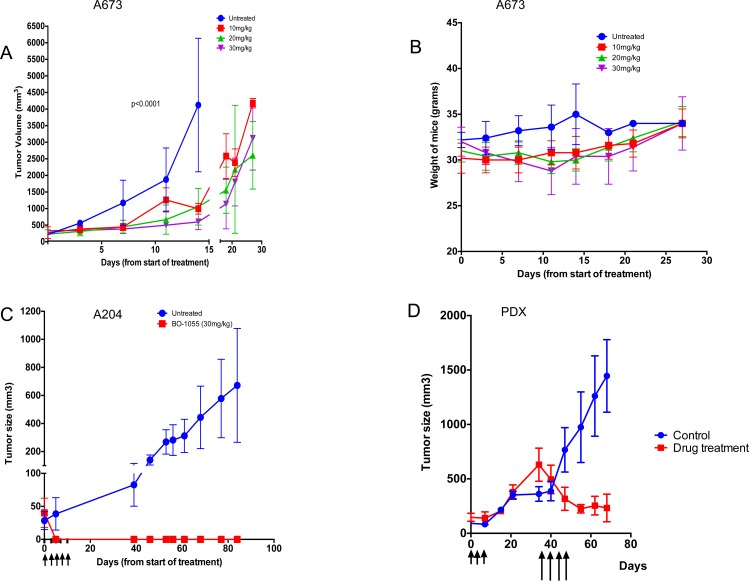
BO-1055 inhibits Ewing sarcoma tumor growth in NSG mice and and causes complete regression of rhabdoid tumor growth in nude mice **A.** NSG mice (*n* = 5 per group) bearing A673 xenografts of approximately 100mm3 size were given 5 doses of BO-1055 at 10mg/kg, 20mg/kg and 30mg/kg doses by tain vein injection. Tumor volume was measured twice a week and plotted. **B.** Weights of NSG mice treated at different doses of BO-1055. **C.** Nude mice (*n* = 5 per group) bearing A204 xenografts were treated with BO-1055 at a dose of 30mg/kg for doses by tail vein inj. Complete regression of tumors were noted in the treated group. **D.** Xenografts in NSG mice were developed from a patient with relapsed Ewing sarcoma. When the tumors reached 100mm2, we randomized mice in to control and drug treatment groups (*n* = 5 per group). Drug treatment group received MTD dose of cyclophosphamide 70mg/kg i.p. q2d for 3 doses (shown by shorter arrows). Tumor growth was not inhibited in the cyclophosphamide treated mice. When the tumors reached above 500mm3, we started treatment with BO-1055 at a dose of 30mg/kg i.v. q2d for 4 doses (shown by longer arrows).

BO-1055 caused inhibition of tumor growth in A673 xenografts (*n* = 20) and prolonged survival when mice were treated with BO-1055 at 10mg/kg, 20mg/kg and 30mg/kg/dose q2d x 5; also mice showed no significant decrease in body weight (Figure 5A, 5B). Complete regression of tumors in nude mice bearing A204 xenografts was noted in the treatment group (Figure 5C) with no significant decrease in body weight. Patient derived xenografts were established from Ewing sarcoma patients who progressed through first line chemotherapy that included cyclophosphamide, doxorubicin, vincristine, ifosfamide and etoposide (PS3). When these Ewing sarcoma xenografts (*n* = 10) reached 100mm^3^, they were randomized to control and treatment groups. When the tumors progressed to 500mm^3^ in the treatment group after receiving MTD doses of cyclophosphamide, the mice were treated with BO-1055 30mg/kg/dose q2d x 4, which resulted in tumor regression (Figure 5D).

### Combination of BO-1055 and irinotecan exhibits synergism against Ewing sarcoma *in vitro* and *in vivo* models

**Figure 6:**
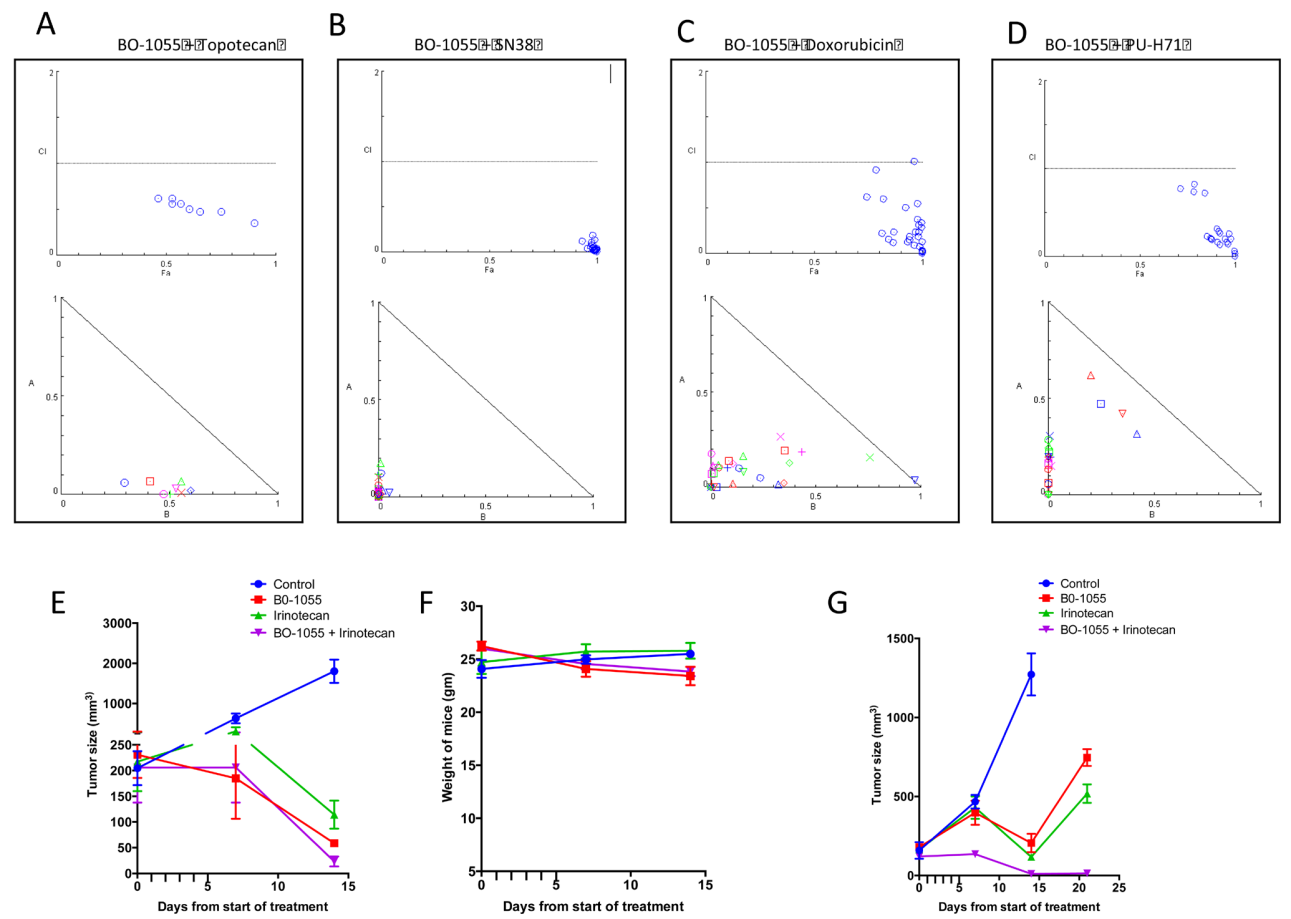
**Combination of A. BO-1055 and topotecan, B.** BO-1055 and SN-38, **C.** BO −1055 and Doxorubicin, **D.** BO-1055 and PU-H71, exhibits synergism against Ewing sarcoma cells *in vitro*. Varying concentrations of BO-1055 and the second drug were applied simultaneously in a lattice format in a 96 well plate and the cytotoxicity was quantified using Alamar blue cell proliferation assay. Fa-combination index (CI) plot and normalized isobolograms were generated using Compusyn software for each combination shown in the figure. Combination Index (CI) < 1 indicated synergism, CI = 1 indicates additive effect and CI > 1 indicates antagonism of various drug combinations, **E.** Patient derived Ewing sarcoma xenografts (PDX) were established in NSG mice (*n* = 20) and they were randomized into 4 groups, the control group received PBS, the BO-1055 group received 10mg/kg IV daily for 5 days, the Irinotecan group received 10mg/kg IP daily for 5 days and the combination group received both the drugs at same dose schedule. **F.** The weight of mice were similar in all the four groups **G.** PDX in NSG mice (*n* = 12) were randomized into 4 groups, the control group received PBS, the BO-1055 group received 5mg/kg IV daily for 5 days, the Irinotecan group received 5mg/kg IP daily for 5 days and the combination group received both the drugs at same dose schedule. Tumor regression was significantly greater in the combination group compared to BO-1055 or Irinotecan groups when measured at day 14 (E, G).

We combined BO-1055 with drugs that are commonly employed in a combination regimen with alkylating agents in varying concentrations of each drug in a lattice format in a 96 well plate. The cytotoxicity to the sarcoma cells was quantified using Alamar blue cell proliferation assay. BO-1055 was noted to be synergistic with SN-38 (active metabolite of irinotecan), topotecan, doxorubicin and PU-H71 (a novel HSP90 inhibitor). Fa-combination index (CI) plot and normalized isobolograms were generated using Compusyn software for each combination (Figure 6A-6D). Patient derived Ewing sarcoma xenografts were established in NSG mice and they were randomized into 4 groups as mentioned in the methods section. Tumor regression was significantly greater in the combination group compared to BO-1055 or Irinotecan groups (given at 10mg/kg/dose qd x 5 or 5mg/kg/dose qd x 5) when measured at day 14 (Figure 6E, 6G). No significant weight loss was noted in the combination group compared to the groups treated with single drugs (Figure 6F).

## DISCUSSION

Sarcomas account for 10-15% of all pediatric cancers. Overall survival for children with relapsed and metastatic sarcomas is poor and remains unchanged over the past 2 decades [17]. Alkylating agents including cyclophosphamide, ifosfamide and temozolomide are active in various sarcomas and are part of first line and second line chemotherapy regimens [1, 18–21]. There have been ongoing efforts to develop novel alkylating agents to avoid the side effects seen with the standard agents [22–24], however it is yet to be determined if any of them are superior to the earlier agents.

We have developed a series of water-soluble N-mustard-benzene conjugates with potent antitumor activity. Of these derivatives, we previously demonstrated that BO-1055 (Ureidomustine) has a broad spectrum of antitumor activity [6, 25, 26] and has little or no cross resistance to taxol or vinblastine in leukemia cell lines [6]. In the present studies, we further demonstrate that this agent has a wide therapeutic window determined by comparing its cytotoxicity in various benign cells and in sarcoma preclinical models, including tumors that are resistant to cyclophosphamide. BO-1055 is a bi-functional alkylating agent that induces interstrand cross-linking and double stranded breaks leading to G2/M arrest and apoptosis of rapidly proliferating cells [12]. Typically cells respond by activating DNA repair mechanisms to restore integrity of the genome. Cells that have deficient repair mechanisms are exquisitely sensitive to such agents [27]. As shown in Figure 1, some cell lines including osteosarcomas are resistant to BO-1055. It is possible that a variation in DNA damage repair mechanisms could be contributing to resistance in these cell lines. In fact, polymorphisms in DNA repair and glutathione-S-transferase genes were shown to influence treatment outcome in osteosarcoma [28]. Increased sensitivity to DNA damaging agents in Ewing sarcoma can be explained by defective DNA break repair and down regulation of DNA repair genes BRCA1, GEN1, and ATM [29, 30]. We noted an increase in the expression of pATM suggesting activation of ATM mediated DNA repair mechanism in cell lines that were both sensitive and resistant to BO-1055.

BO-1055 exhibited remarkable lack of toxicity in normal benign cells including mesenchymal stromal cells, hematopoietic progenitor cells, and endothelial cells which may indicate an alternative option to the widely used toxic alkylating agents. The findings on comet assay and γ-H2AX expression suggest that BO-1055 induces far less DNA double strand breaks in hMSC and CD34+ hematopoietic cells when compared to A673 Ewing sarcoma cells. A number of pre-clinical and clinical studies have shown that dose escalation of cytotoxic agents resulted in improved cure rates for solid tumors and hematopoietic malignancies [31]. However myelotoxicity has been a challenge, resulting in delay in treatment cycles and infectious complications. Hematopoietic toxicity was partly alleviated by the administration of G-CSF [32] and allowed dose-escalation [33]. Interval compressed cytotoxic chemotherapy administered every 2 weeks as opposed to every 3 weeks has produced superior results in localized Ewing sarcoma [34]. Overcoming hematopoietic and organ toxicities will allow us to administer chemotherapy at a higher dose and at a shorter interval, which can potentially result in better cure rates. BO-1055 exhibited a remarkable therapeutic window for hematopoietic progenitors as noted on CFC assay (Figure 2B). Compared to other DNA damaging agents including 4-HC, etoposide and cisplatin, BO-1055 had a 25-50 fold broader therapeutic window (Figure 2C). This was also seen in the *in vivo* toxicity study in C57 healthy mice where hematologic toxicity was not observed at day 10 after 5 doses of BO-1055 at 30mg/kg/dose (Supplementary Table 1).

As a single agent, suppression of A673 tumor growth was maintained with BO-1055 at 30mg/kg/dose during the course of treatment and significant tumor suppression and prolongation of survival was noted with 10mg/kg, 20mg/kg and 30mg/kg doses in treated mice compared to control mice. In A204 (rhabdoid) tumor bearing mice, BO-1055 caused complete regression of tumors. In a patient derived xenograft model, Ewing sarcoma tumors that were resistant to cyclophosphamide showed regression of tumors with 4 doses of BO-1055, suggesting lack of cross resistance with cyclophosphamide. Acquired resistance to alkylating agents is a frequently encountered clinical problem that results in treatment failure. Cross -resistance to various alkylating agents is an established phenomenon but not seen with all the agents in this class of drugs [35, 36]. BO-1055 was shown to retain cytotoxicity against CCRF-CEM/Taxol cells, which are 330-fold resistant to taxol. In comparison, BO-1055 has only a 9.4 fold resistance to CCRF-CEM/Taxol, suggesting that BO-1055 is not a good substrate of membrane multidrug resistance transporters including p-glycoprotein [6]. Cytotoxic activity in PGP-expressing cell lines and in cyclophosphamide resistant xenografts suggests that BO-1055 may not share the same resistance mechanisms with other agents.

DNA damaging agents have been shown to have synergistic effects with a number of drugs based on cell cycle specific actions, topoisomerase inhibition and induction of cellular stress response. We therefore tested four combinations including topotecan, SN38, doxorubicin and PU-H71. We noted synergism with each of these drugs, but the combination with SN38 was highly synergistic. An *in vivo* experiment in NSG mice bearing patient-derived Ewing sarcoma xenografts using Irinotecan and BO-1055 showed significantly greater tumor regression compared to single drugs, suggesting a potential utility of this combination for maximal anti-tumor activity. Synergism between DNA cross-linking agents and topoisomerase 1 inhibitors has been suggested in other cancer models, partly due to increased retention of DNA interstrand crosslinks [37–39]. Recent clinical trials that evaluated a combination of irinotecan and alkylating agents such as temozolomide have proven to be safe and effective in pediatric solid tumors [19, 40, 41]. The pharmacokinetics of BO-1055 has been studied in rats using high-performance liquid chromatography with photodiode array (HPLC-PDA) method suggesting an acceptable PK profile with rapid distribution to all organs except brain and a slow elimination of the drug [7]. We noticed that certain cell lines and tumor types e.g. osteosarcoma were resistant to BO-1055. Further elucidation of mechanisms of resistance and biomarkers for predicting the sensitivity cell lines to BO-1055 is being studied. Superior single agent activity, minimal toxicity to benign cells, lack of cross resistance, synergism with topoisomerase inhibitors and favorable pharmacokinetics are some of the promising attributes that warrant clinical evaluation of BO-1055 in pediatric sarcomas.

## MATERIALS AND METHODS

### Cells lines and patient samples

Ewing sarcoma cell line A673, Prostate (PC-3, LNCaP, 22RV/HL2), colon (HCT-116), breast (MCF-7, MX-1), small cell lung cancer (H526) and renal (A-498, 786-0) cancer cell lines were purchased from American Type Culture Collection (ATCC, Manassas, VA). Dr. Marc Ladanyi (Department of Pathology, MSKCC) kindly provided the following cell lines: CHLA-9, TC32, RMS559 and TE-381. Dr. Gary Schwartz kindly provided the following osteosarcoma cell lines: OSA, U20S and SAOS. Dr. Constantine Markides, (CHRISTUS Stehlin Foundation for Cancer Research, TX) kindly provided Ewing sarcoma and desmoplastic small round cell tumor (DSRCT) cell lines, including CAR, ZUC, ORA, BER and BOD. Early cultures derived from Ewing sarcoma patient samples, including IARC-EW1, SIM-1 were obtained from the MSKCC monoclonal antibody core facility. STR testing done by Genetic Resources Core Facility at Johns Hopkins University, Baltimore, Maryland, authenticated preexisting cell lines. The Monoclonal Antibody Core Facility at MSKCC tested all cell lines for mycoplasma contamination. Cells were maintained in a 37^0^ C, 5% CO2, fully humidified incubator. Ewing sarcoma cell lines A673, CHLA-9, TC32, CAR, ZUC, ORA, IARC-EW1 and SIM-1 and DSRCT cell lines BER, BOD were grown in Dulbecco's Modified Eagle's Medium and supplemented with 10% Fetal Bovine Serum (FBS) (Atlas Biologicals, Fort Collins, CO), L-glutamate and antibiotics penicillin/streptomycin (Gibco, Grand Island, NY). Osteosarcoma cell lines, OSA, U2OS, SAOS were cultured in McCoy's medium (Gibco) and rhabdomyosarcoma cell lines, RH30, TE-381, A204 were cultured in RPMI medium (Gibco) supplemented with 10% FBS, L-glutamate and antibiotics penicillin/streptomycin. In all experiments, cells were plated in 6, 12 or 96-well plates 12 hours (h) before treatment. Benign cells including IMR-90 (fetal lung myofibroblasts) and HS27 (adult human BM mesenchymal cells) were purchased from ATCC; Dr. Shahin Rafii (Cornell University) kindly provided HUVEC (human umbilical vein endothelial cells); FBMC (fetal bone marrow mesenchymal cells), MSC (mesenchymal stromal cells) and BMMSC (adult human BM derived mesenchymal cells), and BMEC-hTert-immortalized bone marrow-derived microvascular endothelial cells were obtained from Moore laboratory, MSKCC. Human umbilical cord blood (CB) was purchased from the New York Blood Center. Human CD34+ cells were isolated from Ficoll-separated mononuclear CB cells using the MACS CD34 isolation kit (Miltenyi Biotech, Auburn, CA). De-identified patient tumor tissues were obtained in accordance with and having approval from the MSKCC institutional review board (IRB). Written consent was obtained from the patients prior to sample collection. These patients were treated with chemotherapy including cyclophosphamide, vincristine and doxorubicin prior to tumor resection. We obtained single cell suspensions by physical disruption and digestion of tissues using collagenase type IV (Gibco, Grand Island, NY).

### Drugs and chemicals

BO-1055 was synthesized at Dr. Tsann Long Su's laboratory at the Institute of Biomedical Sciences (IBMS), Academia Sinica, Taipei, Taiwan. PU-H71 was synthesized at Dr. Gabriela Chiosis' laboratory at Memorial Sloan-Kettering Cancer Center (MSKCC), New York, NY, USA. Topotecan was purchased from Sigma-Aldrich (Saint Louis, MO), etoposide, melphalan and 4-hydroperoxy cyclophosphamide (4-HC) were purchased from Santa Cruz Biotechnology (Dallas, TX). SN38, Bendamustine hydrochloride, vincristine sulfate and cisplatin were purchased from Tocris (Bio-Techne, Minneapolis, MN). Doxorubicin and cyclophosphamide were purchased from the pharmacy at MSKCC.

### Assessment of cell proliferation

AlamarBlue^®^ assay (Invitrogen, Carlsbad, CA, USA) was performed to evaluate anti-proliferative activity of the drugs in cell lines and primary cells. Cells were plated in 96-well plates (5×10^5^ cells/well in 200 μL of medium). After 12 h, one of the previously mentioned drugs was added to each well at the specified concentration and incubated for 72 h. At the end of the incubation period, 20 μL of stock solution (0.312 mg/mL) of the Alamar Blue was added to each well. The absorbance was measured using the Synergy H1 hybrid multi-mode microplate reader (BioTek, USA). The drug effect was quantified as the percentage of control absorbance at 540 nm and 585 nm. Optical density was determined for 3 replicates per treatment condition and cell proliferation in drug-treated cells was normalized to their respective controls. All experiments were performed in triplicate.

### Caspase 3/7 activity

A673 cells were plated at a density of 5 × 10^3^ cells/well in 96 well plates. After overnight incubation, BO-1055 was added at specified concentrations as shown in Figure 3C. At 48h and 72h post drug exposure, 100μl of Caspase-Glo 3/7 reagent provided in the ApoTox -Glo Triplex Assay (Promega, USA) was added as per the protocol and the luminescence, which indicates caspase activation, was measured using the Synergy H1 hybrid microplate reader.

### Colony forming unit assay (CFU) assay

Colony assays were performed in triplicate in 24 well plates using 1.2% methylcellulose (Dow Chemical, Waterloo, NY), 30% FCS, 57.2 μM β-mercaptoethanol, 2 mM glutamine, 0.5 mM hemin (Sigma, USA), 20 ng/mL interleukin 3 (IL-3), granulocyte colony stimulating factor (G-CSF), c-KIT ligand (KL) and 6 U/mL erythropoietin (Epo). IL-3 and KL and Epo were obtained from R and D Systems (Minneapolis, USA) and G-CSF was from Amgen (Thousand Oaks, CA). Colonies were scored 14 days after plating as per the protocol described earlier [42].

### Spheroid assay in 3D culture

A673 cells were seeded on flat bottom non attachment 24 well plates (1000 cells/well) in triplicate using 1.2 % methylcellulose (Dow Chemical, Waterloo, NY) mixed with 20% FCS and specified concentrations of BO-1055 or 4HC as shown in Figure 1D. Samples were incubated at 37°C / 5 % CO_2_ and colonies were scored 14 days after plating.

### Flow cytometry

Cell viability and apoptosis were determined using 7-AAD (BD Pharmingen, San Diego, CA) and Annexin V-APC (BD Pharmingen, San Diego, CA) staining according to the instructions provided by the manufacturer and as previously published [43–45]. Cell cycle fractions were determined by propidium iodide nuclear staining. Briefly, cells were harvested, washed in PBS, fixed with 70% ethanol, and incubated with propidium iodide/RNase buffer (BD Biosciences, San Diego, CA) for 15 minutes at room temperature. Data was collected on BD LSR Fortessa fluorescence-activated cell analyzer using BD FACS Diva software and analyzed using FlowJo version 9.6 software (Tree Star, Inc. Ashland, OR). Cell cycle analysis was done by applying the Dean/Jett/Fox cell cycle model using FlowJo software.

### Measurement of DNA damage using comet assay

Comet assay was performed according to the protocol described earlier [15]. Cells were treated with BO-1055 at 2μM concentration for 3h, or immediately after exposure to 8Gy radiation after which they were isolated and mixed with 1% low melting point agarose in a tube. The cells in agarose were transferred to slides coated with normal melting point agarose. The agarose-immobilized cells were lysed in alkaline solution and the agarose trapped DNA was electrophoresed. DNA was stained with ethidium bromide and image acquisition was done immediately. DNA damage quantification was performed using using OpenComet v1.3, an open-source software plugin in Image J 1.47v software (NIH, USA) for automated analysis. [29] Olive moment, which is the product of tail DNA% and the distance between the intensity-weighted centroids of head and tail was taken from the output for analysis. [46]

### Determination of γ-H2AX expression in cells

Staining of γ-H2AX antigen was carried out following the manufacturer's protocol. Briefly, human sarcoma cells and human mesenchymal stem cells were treated with various concentrations of BO-1055 at 37°C. At 3h, the cultures were washed with 1 ml of cold PBS three times, detached with 0.05% trypsin, and cells were pelleted. The pelleted cells were resuspended with 0.2 ml Cytofix/Cytoperm solution (BD Bioscience), incubated at 4°C for 20 minutes, and then washed with 1 ml cold Perm/Wash solution (BD Bioscience) three times. After anti-human γ-H2AX antibody (BD Bioscience) staining, the specimens were subjected to flow cytometric analysis via a Calibur flow instrument (BD Bioscience).

### Immunoblot analyses

Protein concentrations were determined using the BCA kit (Pierce Biotechnology, Rockford, IL) according to the manufacturer's instructions. Protein lysates (20-100 μg) were electrophoretically resolved by SDS/PAGE, transferred to nitrocellulose membrane, and probed with the indicated primary antibodies: Phospho-ATM (Ser1981) (D6H9) from rabbit (1:500, 5883, Cell Signaling, Danvers, MA), Phospho-BRCA1 (Ser1524) from rabbit (1:500, 9009, Cell Signaling), DNA-PK from rabbit (1:500, 4602, Cell Signaling), Mre11 (31H4) from rabbit (1:500, 4847, Cell Signaling) and Rad50 from rabbit (1:500, 3427, Cell Signaling). Membranes were then incubated with a 1:5,000 dilution of a peroxidase conjugated corresponding secondary antibody. Equal loading of the protein samples was confirmed by parallel western blots for β-actin (1:5,000, ab822750; Abcam). Detection was performed using the ECL-Enhanced Chemiluminescence Detection System (GE Healthcare Biosciences, Pittsburgh, PA) according to the manufacturer's instructions. Blots were visualized by autoradiography.

### Assessment of tumor progression in sarcoma *in vivo* model

All mouse experiments were performed at the animal core facility at MSKCC under an approved protocol following the IACUC guidelines. Five to seven-week-old NOD/SCID IL2R gamma null (NSG) mice (bred at the Mouse Genetics Core Facility, MSKCC) were used for *in vivo* experiments. All mouse experiments were done following the IACUC guidelines at the animal facility at MSKCC. Tumors were induced using 10^6^ A673 cells or A204 cells in 0.2ml 1:1 cellular medium, Matrigel matrix (BD Bioscience, Bedford, MA) and injected subcutaneously (s.q) into the right flank. The tumors were measured every 3 d with a caliper, and the diameters were recorded. Tumor volume was calculated by the formula a^2^ b/2 where ‘a’ is the smallest and ‘b’ is the largest diameter. When the tumors reached 100mm^3^, we randomized mice into control and drug treatment groups. Treatment groups received BO-1055 by tail vein injection at 10mg/kg, 20mg/kg and 30mg/kg q2d x 5 doses for mice bearing A673 xenografts. BO-1055 was given at 30mg/kg q2d x 5 doses for mice bearing A204 xenografts. De-identified patient tumor tissues were obtained in accordance with and approval from the MSKCC Institutional Review Board (IRB). Appropriate written consent and assent were obtained from the patients prior to sample collection. Patient derived xenografts were developed in NSG mice (second passage) from a patient with relapsed Ewing sarcoma, who was treated with two alkylating agents- cyclophosphamide and ifosfamide as part of a multi-agent chemotherapy prior to relapse. When the tumors reached 100mm^2^, we randomized mice into control and drug treatment groups. Mice in the control groups received PBS injections while mice in the drug treatment group received the maximum tolerated dose (MTD) of cyclophosphamide 70mg/kg i.p. q2d x 3 (shown by shorter arrows) (Figure 5D). Tumor growth was not inhibited in the cyclophosphamide treated mice. When the tumors reached a size greater than 500mm^3^, we started treatment with BO-1055 at a dose of 30mg/kg i.v. q2d x 4 doses (shown by longer arrows in Figure 4F). For the combination *in vivo* study, we used PDX from Ewing sarcoma patients that were established in NSG mice (*n* = 12-20 per PDX model) and randomized into 4 groups: the Control group received PBS, the BO-1055 group received 5 mg/kg in one PDX model and 10mg/kg in another PDX model IV daily for 5 days, the Irinotecan group received 5 mg/kg in one PDX model and 10mg/kg in another PDX model IP daily for 5 days and the combination groups received both the drugs at the same dose schedule.

### Drug combination studies

The AlamarBlue^®^ cell proliferation assay was used to test the effect of a single drug and a combination of drugs. A673 cells (5 × 10^3^ cells/well) were added to 96 well plates in triplicate. After 12h incubation, BO-1055 and a second drug (Topotecan, SN38, Doxorubicin or PU-H71) were added simultaneously in a lattice format in varying concentrations. Plates were read after 72h drug exposure. Combination indices were derived as described below to identify the interaction in two-drug combinations.

### Statistical analysis

Data are presented as mean±S.E.M. of at least three independent experiments done in triplicate. The comparison of means between treated and control mice was performed using two-tailed *T*-test or ANOVA (for more than two groups) as implemented in GraphPad Prism (version 4; GraphPad Software). Graphpad Prism software was used to plot concentration-effect curves and determine the drug concentration that inhibited the growth of cell lines by 50% compared to the control (IC_50_). A p-value of 0.05 or less was considered significant. Combination index (CI) values, as calculated by CompuSyn software (Chou and Martin, 2007), were used to evaluate the interaction between BO-1055 and a second drug (Topotecan, SN38, Doxorubicin or PU-H71). The Chou-Talalay method was used to quantify the synergism (CI < 1), additive effect (CI = 1) and antagonism (CI > 1) of various drug combinations [47].

## SUPPLEMENTARY MATERIAL


