# Supplementary material for: BO-1055, a novel DNA cross-linking agent with remarkable low myelotoxicity shows potent activity in sarcoma models
Source: Oncotarget. 2016 May 29;7(28):43062–75. doi: 10.18632/oncotarget.9657 (PMC5190008; doi:10.18632/oncotarget.9657)
Supplement: Supplementary file 1 [file oncotarget-07-43062-s001.pdf]

BO-1055, a novel DNA cross-linking agent with remarkable low myelotoxicity shows potent activity in sarcoma models

Supplementary Material

Supplementary table 1. Complete blood counts, renal and kidney function tests in C57 healthy mice (n=4 per group) in control and treatment groups. Treatment group received BO-1055 at 30mg/kg/dose on days 1,3,5,8 and 10.

	Control group baseline			BO-1055 group baseline			Control group day 10			BO-1055 group day 10	
	Mean	SD		Mean	SD		Mean	SD		Mean	SD
RBC (M/uL)	9.62	0.94		9.58	0.42		9.22	0.23		9.08	0.36
HGB (g/dL)	13.90	1.04		14.00	0.36		13.50	0.22		13.08	0.50
HCT (%)	48.03	3.88		48.43	1.00		47.73	1.31		46.30	2.07
MCV (fL)	50.00	1.02		50.57	1.79		51.75	0.54		51.03	2.51
MCH (pg)	14.47	0.40		14.60	0.44		14.65	0.21		14.40	0.29
MCHC (g/dL)	28.93	0.57		28.90	0.20		28.30	0.35		28.28	0.84
RDW-SD (fL)	32.57	1.14		33.20	1.15		33.95	1.38		32.48	0.57
RDW-CV (%)	24.37	1.11		24.23	1.93		23.98	0.89		23.18	1.28
RET# (K/uL)	404.70	11.65		485.50	146.00		419.35	20.03		426.88	51.84
RET (%)	4.23	0.37		5.11	1.74		4.55	0.20		4.72	0.74
PLT (K/uL)	908.70	309.90		844.30	314.00		1122.50	76.75		1108.25	152.17
PDW (fL)	7.20	0.44		7.07	0.72		6.65	0.10		7.40	0.49
MPV (fL)	6.30	0.30		6.17	0.38		6.05	0.19		6.38	0.17
WBC# (K/uL)	8.63	2.26		13.60	1.66		8.13	1.53		5.34	1.98
NEUT# (K/uL)	0.86	0.17		1.92	0.80		1.66	0.60		1.90	0.75
LYMPH# (K/uL)	7.55	2.10		11.34	2.36		6.21	1.34		2.90	1.83
MONO# (K/uL)	0.06	0.04		0.09	0.03		0.16	0.09		0.39	0.12
EO# (K/uL)	0.15	0.03		0.24	0.05		0.08	0.04		0.13	0.07
BASO# (K/uL)	0.01	0.01		0.01	0.01		0.03	0.02		0.02	0.02
NEUT (%)	10.07	0.87		14.70	7.92		20.48	7.23		37.20	14.23
LYMPH (%)	87.23	1.36		82.77	8.39		76.45	7.20		51.63	14.63
MONO (%)	0.73	0.29		0.67	0.12		1.83	0.77		7.80	2.58
EO (%)	1.87	0.68		1.80	0.62		0.95	0.37		2.93	2.35
BASO (%)	0.10	0.10		0.07	0.06		0.30	0.16		0.45	0.31
BUN (mg/dL)	31.25	2.50		37.50	5.45		31.50	3.87		24.50	5.26
CREA (mg/dL)	0.20	0.03		0.23	0.04		0.22	0.03		0.20	0.03
BUN/CREA ratio	159.53	12.67		166.35	36.62		143.80	13.46		125.40	28.03
ALT (U/L)	48.75	9.00		78.50	22.71		79.50	32.73		89.50	45.83
AST (U/L)	50.50	5.51		69.25	12.42		63.00	13.90		74.25	26.89
TBIL (mg/dL)	0.20	0.00		0.25	0.06		0.13	0.05		0.23	0.19
DBIL (mg/dL)	0.00	0.00		0.05	0.10		0.00	0.00		0.00	0.00
IBIL (mg/dL)	0.20	0.00		0.20	0.14		0.13	0.05		0.13	0.06
TP (g/dL)	5.20	0.08		4.08	1.73		3.68	0.97		3.77	0.49
GLU (mg/dL)	293.00	57.56		218.00	84.06		211.25	94.21		166.00	41.33
